# Endocrine Fertility Parameters—Genomic Background and Their Genetic Relationship to Boar Taint in German Landrace and Large White

**DOI:** 10.3390/ani11010231

**Published:** 2021-01-18

**Authors:** Ines Brinke, Christine Große-Brinkhaus, Katharina Roth, Maren Julia Pröll-Cornelissen, Sebastian Klein, Karl Schellander, Ernst Tholen

**Affiliations:** 1 Institute of Animal Science, University of Bonn, 53115 Bonn, Germany; ibri@itw.uni-bonn.de (I.B.); krot@itw.uni-bonn.de (K.R.); mpro@itw.uni-bonn.de (M.J.P.-C.); ksch@itw.uni-bonn.de (K.S.); etho@itw.uni-bonn.de (E.T.); 2 Association for Bioeconomy Research (FBF e.V.), Adenauerallee 174, 53113 Bonn, Germany; ks@fbf-forschung.de

**Keywords:** boar taint, fertility, hormones, pigs, androstenone, skatole, cortisol, estradiol, testosterone, luteinizing hormone, follicle-stimulating hormone

## Abstract

**Simple Summary:**

Breeding against boar taint compounds androstenone and skatole can be an efficient alternative for surgical castration of young piglets during their first week of life to avoid boar taint. Physiological links of androstenone to steroid hormones in the synthesis pathway are documented and they have to be analyzed for their genetic effects on reproduction and fertility. While using boar taint and hormone data from Landrace and Large White pigs (commercial nucleus populations and herd book populations), the effects of breeding against androstenone on fertility were evaluated. Moreover, the genetic foundation of the chosen hormones’ testosterone, 17β-estradiol, luteinizing hormone, follicle-stimulating hormone, and progesterone was analyzed and then checked for possible pleiotropic effects with boar taint compounds. The results showed consistent unfavorable side effects of breeding against androstenone on testosterone and 17β-estradiol in both breeds. The other hormones showed contrary results regarding unfavorable relationships between boar taint and endocrine fertility parameters. The genetic foundation showed a high potential of breeding against boar taint but the impact on fertility potential should be supervised.

**Abstract:**

The surgical castration of young male piglets without anesthesia is no longer allowed in Germany from 2021. One alternative is breeding against boar taint, but shared synthesis pathways of androstenone (AND) and several endocrine fertility parameters (EFP) indicate a risk of decreasing fertility. The objective of this study was to investigate the genetic background between AND, skatole (SKA), and six EFP in purebred Landrace (LR) and Large White (LW) populations. The animals were clustered according to their genetic relatedness because of their different origins. Estimated heritabilities (h^2^) of AND and SKA ranged between 0.52 and 0.34 in LR and LW. For EFP, h^2^ differed between the breeds except for follicle-stimulating hormone (FSH) (h^2^: 0.28–0.37). Both of the breeds showed unfavorable relationships between AND and testosterone, 17-β estradiol, and FSH. The genetic relationships (r_g_) between SKA and EFP differed between the breeds. A genome-wide association analysis revealed 48 significant associations and confirmed a region for SKA on *Sus Scrofa* chromosome (SSC) 14. For EFP, the results differed between the clusters. In conclusion, r_g_ partly confirmed physiologically expected antagonisms between AND and EFP. Particular attention should be spent on fertility traits that are based on EFP when breeding against boar taint to balance the genetic progress in both of the trait complexes.

## 1. Introduction

The breeding objectives of most pig breeding organizations try to balance economically important production, reproduction, and fitness traits, including animal welfare and meat quality aspects. In the past (<2010), these breeding goals were dominated by traits, like fat and carcass composition characteristics, as well as litter size [1]. As a consequence of intensive discussion with society and consumers, increased attention has recently been paid to animal welfare aspects. This leads to changes in the breeding objective in favor of traits, like prenatal survival, vitality, uniformity of the litter, and robustness, which are becoming as important as litter size [1].

According to the German animal protection law, the castration of piglets without anesthesia is banned from the year 2021 [2]. Because of this legal regulation, the fattening of entire boars has been an attractive alternative, not only because of animal welfare reasons, but also because of improved sustainability of pig production regarding feed conversion rate, carcass composition [3], and carbon footprint [4]. However, piglets were castrated because of the risk of boar taint, an odorous smell of heated pork meat, due to the onset of puberty. In order to lower the hazard of tainted carcasses, some breeding organizations have extended their performance recording scheme and their breeding goals by boar taint traits. Some of the main causes for the occurrence of boar taint are the sex steroid hormone AND, which is built in the Leydig cells in the testis, and SKA, which is a product of degradation processes of the amino acid tryptophan in the colon [5]. Whereas the production of AND is initiated by puberty [6] and it is mainly affected by genetics, the production of SKA is less determined by genetics but more affected by management and nutrition [5,7,8,9]. Although the origin of these compounds is quite different, there is a moderate positive relationship (genetic correlation (r_g_) = 0.27−0.57 [10,11,12,13]) between AND and SKA concentration in backfat. Doran et al. [14] explained this by the decreased degradation of SKA in the liver metabolism during the presence of a high AND concentration.

For a sustainable prevention of boar taint, breeding against the boar taint compounds is one suitable alternative. Before implementing AND and SKA in selection strategies, possible interactions with other trait complexes, like fertility, have to be investigated in order to avoid loss of breeding progress. Selection for boar taint might have consequences on paternal as well as on maternal fertility. Along with direct reproduction traits, like number of piglets born alive, hormone profiles of sows are a valuable and precise source of information for maternal fertility. In this regard, estimated r_g_ between hormones of the sow as an indicator of maternal fertility and boar taint are useful for characterizing the consequences of selection against boar taint. Previous studies [15,16,17] showed contrary results regarding the genetic interaction between boar taint and male/female fertility. Until now, an impairment of the previous breeding progress in the reproduction traits could not be excluded, due to the fact that steroid hormones and AND are built in the same synthesis pathway of the steroidogenesis. Nevertheless, some studies are based on routinely collected traits, like litter size from commercial breeding organizations, which might not allow intensive insight into the linked hormonal regulation of boar taint, fertility, and robustness.

In order to uncover this complex regulation, we have recorded the hormone profiles of sows and boars that originate from maternal breeding lines of commercial breeding organizations. We focused on the relationship between boar taint compounds and the synthesis of steroid hormones due to the shared synthesis pathway of AND and steroid hormones linked to reproduction [18,19]. Therefore, AND and SKA, as well as testosterone, estradiol, luteinizing hormone (LH), follicle-stimulating hormone (FSH), progesterone, and cortisol were analyzed, due to their relatedness to boar taint or male/female fertility and robustness. The objective of this study was to investigate the possible unfavorable genetic relationships between boar taint and the six steroid hormones by variance component estimation (VCE). Additionally, the quantitative trait loci (QTL) for these traits should be identified in male and female Landrace (LR) and Large White (LW) populations from commercial breeding and herd book organizations in order to clarify possible pleiotropic genetic backgrounds.

## 2. Materials and Methods

### 2.1. Phenotypes

All of the phenotypes that are related to boar taint compounds or hormone profiles were recorded within the LR and LW nucleus populations of a commercial breeding organization (C) and a consortium of four herd book organizations (H), respectively. In the next sections, these data sets were designated as LR_C, LW_C (commercial breeding organization), and LR_H, LW_H (herd book organizations). For all animals, pedigree information was available up to 15 generations.

### 2.2. Boar Taint

A total of 3775 boars were selected within the LR and LW nucleus population of both breeding organizations (C, H). These boars should cover the genetic variability of the LR and LW populations and they were on-station performance tested. LR_C/LW_C boars (n = 1392/1377) were slaughtered at a constant age (~160 days), LR_H/LW_H boars (n = 744/262) at a constant weight (LR_H: 93.4 kg, LW_H: 92.6 kg) in different commercial, EU-certificated abattoirs that were connected to the participating breeding organizations. Adipose tissue samples from these boars were collected from the neck area of the carcasses 24 h after slaughtering and they were stored at −20 °C until analysis. AND and SKA concentration in adipose tissue was analyzed in all of the samples using a standardized stable isotope dilution analysis-headspace solid-phase microextraction-gas chromatography/mass spectrometry (SIDA-HSPM-GC/MS) [20]. Concentrations were log-transformed for further analyses because of the skewness of AND and SKA.

### 2.3. Hormone Profiling

The blood collection was performed in accordance with an approval of the German animal protection law and the regulations for the use of animals (reference number: 84-02.04.2016.A541). The samples were taken from a subset of 500 full-sib pairs (male and female) almost equally distributed across C (n = 252) and H (n = 248). Full-sib pairs consisted of one stationary tested boar out of the boar dataset mentioned above and one sister. Blood samples in female pigs were collected at farms at a live weight of 65 to 75 kg (126.37 days on average ± 13.39). This weight range was chosen in order to characterize the hormone status of the sows before puberty. With the same motivation, male pigs were blood sampled at the station one week before slaughter (164.81 days on average ± 10.7).

For blood collection, animals were fixed (<1.5 min) and approximately 10 mL of blood was taken from the jugular vein. After clotting at room temperature, the serum was separated from each blood sample by centrifugation at 1500× *g* for 10 min. The serum was stored at −80 °C until assay.

Concentrations of luteinizing hormone (LH) and FSH were measured while using competitive ELISA Kits (Pig Follicle Stimulating Hormone (FSH) ELISA Kit; Luteinizing Hormone (LH) ELISA Kit, Abbexa Ltd., Cambridge, UK). The minimum detectable concentrations while using these assays were 0.469 ng/mL for LH and 14.1 ng/mL for FSH. The blood samples from male animals were diluted to 1:5 for measurements of LH, according to the results of preliminary tests. All of the blood samples (from male and female animals) were diluted to 1:10 for measurements of FSH. Cortisol (CORT), progesterone (PROG), 17-β-estradiol (EST), and testosterone (TEST) concentrations were measured while using a Multi-Species Hormone Magnetic Bead Panel (MSHMAG-21K-04 Multi-Species Mag Panel, Merck Chemicals GmbH, Darmstadt, Germany). The minimum detectable concentrations using this assay were 0.17 ng/mL for CORT, 0.14 ng/mL for PROG, 0.01 ng/mL for EST, and 0.08 ng/mL for TEST. All of the values under the sensitivity threshold (censored data) were replaced by half of the minimum detectable value, as described by Hornung and Reed [21]. Measurements of PROG for both sexes were removed for further analyses due to the high number of animals with censored data. All of the analyses were analyzed twice. Samples with an intra-variation coefficient (intra-CV) > 30% were excluded from further analyses due to the quality standards of the corresponding analysis protocols. Details on the number of investigated animals with hormone profiles per breed and sex can be found in the results section. The resulting concentrations were log-transformed due to their skewed distribution. A control sample was run on every plate in order to control the measurement quality. It consists of pooled blood serum samples from seven full-sib boars of one litter. The inter-assay coefficients of variation for the control sample varied between 16 and 20%, depending on the analyzed hormone.

### 2.4. Statistical Analyses

Variance components were estimated with a multivariate approach while using WOMBAT [22] across and within the breeds LR and LW. The analyzed traits were AND, SKA, CORT, EST, TEST, LH, and FSH. In step 1, the hormone profiles of male and female animals were treated as different traits. This can be justified by the different ages at blood sampling and the resulting different stages of maturation of both sexes. In the case of genetic correlation between corresponding hormones of sows and boars close to one we were motivated to see hormones of both sexes as identical traits (step 2). Step 1 was only possible for animals of the LR population, as the sample size of the LW population was too small to analyze the data by using the means of a sex-specific multi-trait model.

For each hormone in LR, the analysis was performed in a four-trait model, which combined pedigree information with the boar taint compounds AND and SKA and one hormone concentration, measured in males and females, respectively. Variance components for AND and SKA were estimated using the following polygenetic animal model:

Model 1 y = X(1)β + Zα + Zu + e

where y contains the observed traits. The generalized linear mixed model was fitted to AND and SKA and it comprised the fixed environmental effect organization-year-month of slaughter (82 levels in LR, 59 levels in LW) in vector β and animal and litter as random effects in vector α and u. Weight and age at slaughter were handled as covariates (Model 1). For the hormone profiles the fixed effects of the organization-year season of blood sampling (21 levels in LR, 9 levels in LW) and plate were considered. The animal (α) and litter (u) were implemented as random effects.

The age at blood sampling was used as a covariate. X and Z were handled as the incidence matrices.

Taking the size of the dataset and the high standard errors into account, these correlations are only a first rough indicator that a large proportion of overlapping genes are involved in the expression of corresponding hormones in both sexes. Accepting these limitations, further analyses for both breeds were performed in a full multiple seven-trait trait model, including AND and SKA (following Model 1) as well as all hormone concentrations following Model 2, including sex as a fixed effect.

### 2.5. Genotype Data

All of the phenotyped sows and boars (LR: 2276 LR, LW: 1694) were genotyped while using the Illumina PorcineSNP60 BeadChip (Illumina, San Diego, CA, USA). These data were used to perform a univariate genome-wide association analysis (GWAS) for analyzed hormones AND, SKA, CORT, EST, TEST, LH, and FSH.

Single nucleotide polymorphism(s) (SNPs) and individuals with a call-rate of less than 95% and SNPs with a minor allele frequency (MAF) of less than 5% were excluded from further analysis. The quality control was conducted within the GenABEL package [23]. The number of genotyped animals varied per trait and sex due to the removal measured hormone phenotypes with a high intra-CV (>30%), as shown in Table 1.

Within the GWAS, log-transformed concentrations of the analyzed hormones, AND, and SKA were regarded as phenotypes. Genetic distances were displayed by creating a genomic relationship matrix within the R-package GenABEL [23]. Based on these visualized genetic distances, GWAS was separately performed within clusters. A small number of animals (n = 20) from the boar taint dataset could not be clearly classified into a cluster and they were excluded from further analyses.

Based on the results of the VCE, male and female hormones were analyzed as identical traits. An association test was performed within the R-package GenABEL [23]. The hormone data were corrected for sex and organization-year-season of blood sampling as fixed effects and age of blood sampling as covariate. The boar taint data were analyzed under consideration of the fixed effects that were described by Model 1.

Population stratification ranged from 1.009 to 1.72, depending on trait and cluster, so that applying genomic control (GC), as described by Devlin and Roeder [24], was sufficient to correct for possible population stratification while using the following formula:

(1) $$T_{\mathrm{corrected}\;}=\;\frac{T^{2}}{\lambda }$$

whereas T^2^ is the empirical test statistic for each locus by a fast score test or t-test and λ is the value of population stratification. The resulting *p*-values were transformed by Bonferroni correction in order to avoid error accumulation by multiple testing. Markers with an adjusted *p*-value < 0.05 were handled as genome-wide/chromosome-wide significant. Additionally, the variance explained by the single SNP was calculated according to the transformation of the student’s t-distribution into a z-distribution [25] while using the following formula:

(2) $$\mathrm{Var}\left[\%\right]\;=\;\frac{\chi_{1\mathrm{df}}^{2}}{{N\;-\;2\;+\;\chi }_{1\mathrm{df}}^{2}}$$

whereas $\chi_{1\mathrm{df}}^{2}$ is the test statistic of each SNP from GWAS and N the number of animals.

The locations of SNPs for all analyzed traits are in accordance with the recent pig genome sequence SusScrofa 11.1, and the variants are identified according to Ensembl release 95 [26].

## 3. Results

### 3.1. Descriptive Summary

The number of animals, overall means, and standard deviations of log-transformed phenotypes are shown in Table 2, for LR and LW, separately. 

Raw phenotypes can be found in the additional Table S1. Boars of the datasets LR_C and LW_C were slaughtered at an average age of 163.5 ± 5.0 days in LR (165.2 ± 5.7 days in LW) and an average slaughter weight of 95.1 ± 9.5 kg in LR (89.0 ± 8.8 kg in LW). Boars of the datasets LR_H and LW_H were slaughtered at an age of 175.1 ± 13.3 days in LR (174.1 ± 13.1 days in LW) and weight of 93.4 ± 6.5 kg in LR (92.6 ± 5.8 kg in LW), on average.

The blood samples for hormone profiling from gilts and boars were taken at an average age of 126.37 days (±13.39) from gilts and at an average age of 164.81 days (±10.70) from boars, respectively.

The average CORT concentrations in LW animals were higher than in LR animals and higher in males than in females. The average TEST concentrations of male animals were clearly higher than in female animals. In general, the LR dataset showed higher TEST concentrations than the LW dataset. The concentrations of EST in female animals did not differ between the breeds, which is in contrast to the male animals were LR animals showed higher concentrations than LW animals. The results of LH and FSH did not show any clear difference between the breeds. Whereas, concentrations of LH were up to 2.5 times higher in female animals, FSH concentrations were lower in female animals than in male animals.

Regarding the boar taint compounds boars from the LR population showed higher concentration of AND and SKA, as compared to the boars from the LW population.

### 3.2. Variance Component Estimation

In general, estimated heritabilities (h^2^), phenotypic (r_p_), and genetic (r_g_) correlations are based on the log-transformed values of all the parameters and they were not transformed in its original scale. An overview of all the results of the VCE can be found in Table 3. 

Step 1 of the VCE showed moderate to high r_g_ between male and female hormone concentrations in LR in a range from 0.40 to 0.91 (see Table 4).

Therefore, further analyses for both breeds were performed in a full multiple seven-trait trait model, including AND and SKA, as well as all hormone concentrations following Model 1, including sex as a fixed effect, as described in the material and methods section.

Variance component estimation showed moderate to high h^2^ of 0.52 for AND in LR (h^2^ = 0.44 in LW) and 0.40 for SKA in LR (h^2^ = 0.34 in LW) (Table 3). Correlations between AND and SKA were higher on the genetic scale in LW (r_g_ = 0.57) than in LR (r_g_ = 0.42), whereas, on the phenotypic scale, it was vice versa (r_p_ = 0.34 in LR, r_p_ = 0.27 in LW). The h^2^ for CORT was 0.11 in LR and 0.35 in LW. The genetic correlation between CORT and other sex steroid hormones was mostly different within the LR and LW breed. For example, the r_g_ between CORT and TEST was −0.35 in LR, but only −0.03 in LW. Genetic correlations between CORT and EST and CORT and FSH in LR were close to zero, whereas, in LW, these relations were moderate to high negative between −0.26 and −0.57. Consequentially, a lower concentration of CORT would possibly lead to a higher EST and a higher FSH concentration in LW but not in LR.

Regarding the r_g_ between CORT and LH similar estimates were found in both breeds (r_g_ = −0.27 in LR, r_g_ = −0.42 in LW). The negative sign indicates that breeding against CORT concentrations would lead to increasing LH concentrations in both of the breeds.

Heritability estimates for steroid hormones were found in a range of 0.03 and 0.42 and they were mostly different within the breeds LR and LW. Among the LR and LW breed, the estimated h^2^ for TEST, EST, and LH were clearly distinguishable. In LR, h^2^ for TEST and EST was small (h^2^ = 0.03 and h^2^ = 0.09), but, in LW, these estimates were moderate to high (h^2^ = 0.23 and h^2^ = 0.42). A converse breed difference was found for LH, where the h^2^ in LR (h^2^ = 0.28) was seven times higher than in LW (h^2^ = 0.04). For FSH, h^2^ was on a moderate level in both of the breeds (LR: h^2^ = 0.28, LW: h^2^ = 0.37).

Genetic correlations between the steroid hormones were consistent between the breeds and they confirmed the physiological expectation of their common regulation (Table 3).

Regarding the objective of our study, r_g_ between boar taint compounds and CORT, as well as steroid hormones, are of major interest.

r_g_ between AND and CORT was low to moderate negative in LR (r_g_ = −0.18) and low in LW (r_g_ = 0.08), as shown in Table 3. Consequently, breeding against AND would result in a small increase in CORT concentration in LR, whereas the effect in LW would be zero. A more distinct breed difference was observed for the r_g_ between SKA and CORT, which was negative (r_g_ = −0.21) in LR, but moderate positive in LW (r_g_ = 0.38).

The AND and TEST as well as SKA and TEST were highly positive correlated to each other in both breeds. The r_g_ between AND and TEST was 0.62 in LR and 0.83 in LW. A similar high positive r_g_ (=0.93) was estimated between SKA and TEST in LR, whereas this relationship was on a comparably lower level in the LW breed (r_g_ = 0.27). Hence, a decrease in AND and/or SKA concentration would result in a lower concentration of TEST in both breeds.

The estimated r_g_ between AND and EST slightly differed between both of the breeds (r_g_ = 0.49 in LR, r_g_ = 0.46 in LW). The r_g_ between SKA and EST was very high in LR (r_g_ = 0.95), but, in contrast to that, the estimate of this relationship in LW did only differ slightly from zero (r_g_ = 0.03).

Regarding the r_g_ between AND and LH, similar trends were observed for LR and LW with low to moderate positive relations (r_g_ = 0.11 in LR, r_g_ = 0.32 in LW). The genetic relationships between SKA and LH are different for both breeds. In LR, a r_g_ of −0.16 was estimated; in LW, it was the opposite, with an estimated r_g_ of 0.45.

For AND and FSH, a moderate r_g_ of 0.30 was estimated for both of the breeds. In contrast to that, r_g_ between SKA and FSH were on a low level, ranging between 0.01 (LW) and −0.14 (LR).

### 3.3. GWAS

#### 3.3.1. Genetic Structure

**Figure 1:**
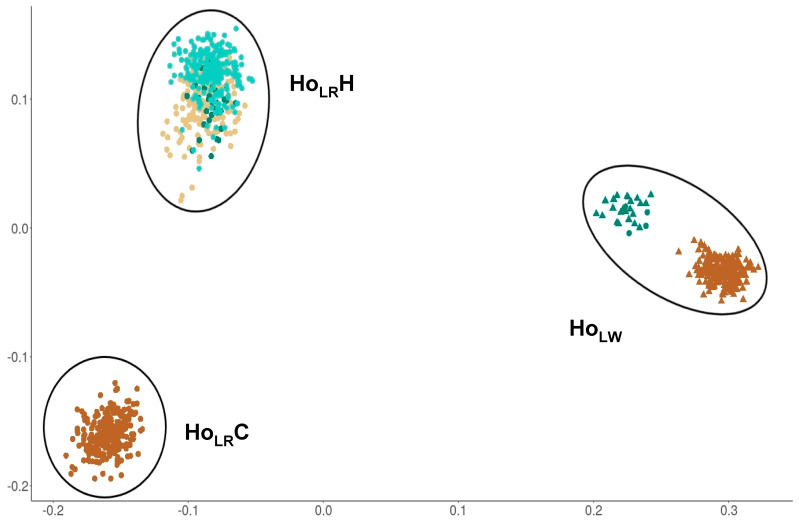
Distribution of animals from hormone dataset in clusters based on genetic relationship matrix, Ho_LR_C n = 254, Ho_LW_ n = 272, Ho_LR_H n = 447. Colors are representing the different organizations. Filled-in circles are representing Landrace animals; filled-in triangles are representing Large White animals.

The visualization of the genetic relationships of all animals showed three different clusters for the hormone dataset (Figure 1). The clusters contain LR animals from the commercial breeding organization (Ho_LR_C), LR animals from the herd book organizations (Ho_LR_H), and LW animals from all organizations (Ho_LW_), whereas the latter one also contains misclassified animals. The number of animals per cluster were 254 animals in Ho_LR_C, 447 animals in Ho_LR_H and 272 animals in Ho_LW_.

**Figure 2:**
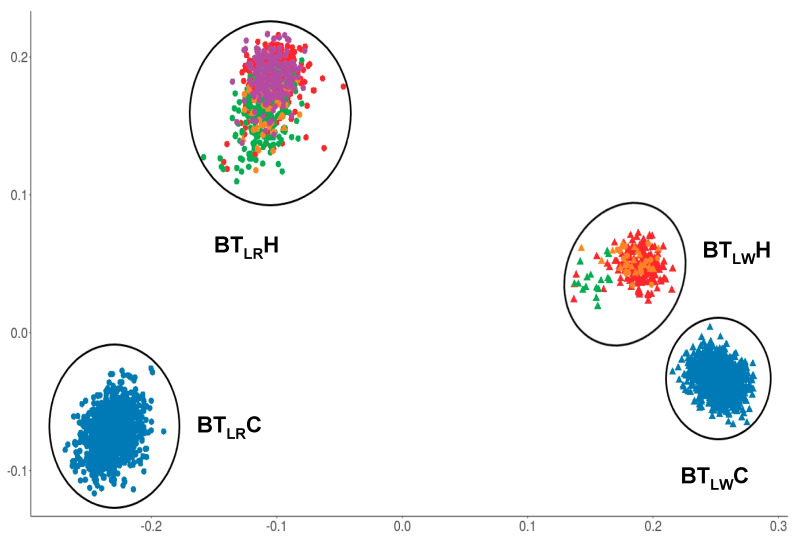
Distribution of animals in clusters based on genetic relationship matrix, BT_LR_C n = 1293, BT_LW_C n = 1317, BT_LW_H n = 256, BT_LR_H n = 735. Colors are representing the different organizations. Filled-in circles are representing Landrace animals, filled-in triangles are representing Large White animals.

For the boar taint dataset, the visualization of the genetic relationships of all animals showed four different clusters (Figure 2). Twenty animals were removed from further analyses, as they cannot be clearly classified into a cluster. Clusters contain LR animals from the commercial breeding organization (BT_LR_C), LW animals from the commercial breeding organization (BT_LW_C), LR animals from the herd book organizations (BT_LR_H), and LW animals from the herd book organizations (BT_LW_H). The number of animals per cluster were 1293 animals in BT_LR_C, 1317 animals in BT_LW_C, 256 in BT_LW_H, and 735 in BT_LR_H.

Cluster BT_LR_C and BT_LW_C are representing LR and LW populations of a commercial breeding organization. GWAS of AND and SKA for these two clusters have already been performed in a previous study [13]. A short summary of the results is presented below. The sample size of cluster BT_LW_H was not sufficient enough for a GWAS (n = 256). Therefore, this study contains an association study of AND and SKA for cluster BT_LR_H, which represents a LR population containing 735 boars of three herd book organizations.

#### 3.3.2. GWAS for Hormone Profiles

Table 5 presents a summary of the genome-wide and chromosome-wide significant associated markers per trait along with their positions for all of the analyzed traits in this study. The full table can be found in the appendix (additional Table S2). A table with adjacent regions and possible interesting genes in a window of ±1 Mb can be found in the additional Table S3. In total, 48 markers were found to be significantly associated with SKA, CORT, TEST, EST, LH, and FSH in the different clusters, whereas no QTL was found for AND. Moreover, there were no overlapping QTL regions across traits or clusters regarding an interval of 2,321,253 basepairs.

CORT was found to be significantly associated with two markers in Cluster Ho_LR_C, nine markers in cluster Ho_LW_ and one marker in cluster Ho_LR_H. In Cluster Ho_LR_C, both of the markers are intron variants and located on SSC 2 at 144.8 Mb. In cluster Ho_LW_, all of the markers were located on SSC 7 in a region from 115.2 to 115.6 Mb, and four of them were also genome-wide significant. Five variants in this region are from a customized chip; therefore, the kind of variant is unknown. The remaining four variants are intron variants. The significantly associated variant in cluster Ho_LR_H is located at 36.1 Mb on SSC 18, but it has not been mapped until now.

For FSH, one intergenic variant at 58.1 Mb on SSC 10 was found to be chromosome-wide significant associated in cluster Ho_LR_C. Additionally, on SSC 7 one intron variant was significantly associated with at 117.9 Mb in cluster Ho_LW_.

For EST and LH, significant markers were only found in cluster Ho_LR_H. Whereas GWAS for LH showed an intron variant as a chromosome-wide significant marker on SSC 7 at 9.5 Mb, GWAS for EST revealed one intron variant on SSC 6 at 69.5 Mb and one non-coding transcript exon variant at 19.6 Mb on SSC 17.

#### 3.3.3. GWAS for AND and SKA

For clusters BT_LR_C and BT_LW_C, which were analyzed in a previous study [13], 25 markers in LR and 18 markers in LW were found to be significantly associated with both of the boar taint compounds. The most important region for AND in LR ranged from 20.9 Mb to 22.9 Mb on SSC 5 and contained 12 significant SNPs. In LW, one marker was found to be chromosome-wide significantly associated with AND at 48.1 Mb on SSC 17. Associations with SKA were found in a shared region for LR and LW on SSC 14 in a region from 140.5 Mb to 141.6 Mb. Additionally, nine markers were found to be chromosome-wide significantly associated with SKA in LR on SSC 6. More details regarding the described regions above can be found in the preceding study by Brinke et al. [13].

**Figure 3:**
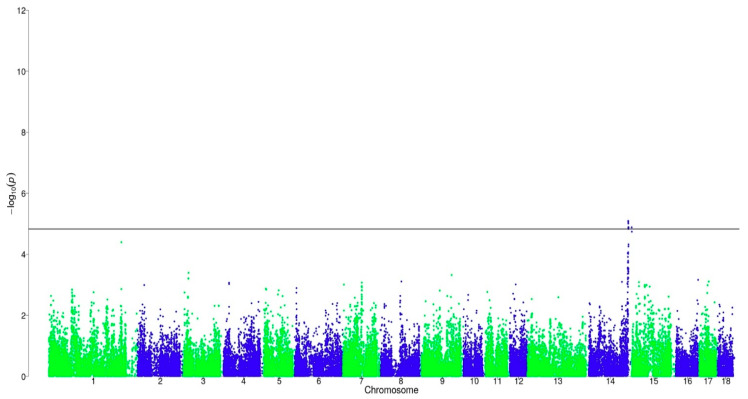
Distribution of SNPs for log-transformed skatole in BT_LR_H. The black line corresponds to the threshold of chromosome-wide significance.

For Cluster BT_LR_H, as analyzed in this study, no significant markers were found to be associated with AND. For SKA, 12 markers were found to be chromosome-wide significantly associated on SSC 14 in a region from 140.5 to 141.3 Mb (Figure 3). It contains four introns, three upstream gene variants, two downstream gene variants, two 3’ prime untranslated region variants, and one intergenic variant. Ten of these markers were also found to be significantly associated with SKA in Cluster BT_LW_C in the previous study [13]. The phenotypic variance that was explained by a significant SNP in this region varied between 3.3% and 3.5%. Additionally, one significant marker was located at 152.9 Mb, which has not been mapped until now.

## 4. Discussion

Breeding against boar taint is a long-term, sustainable, and animal welfare-friendly alternative to surgical castration of young male piglets during their first week of life. Because unfavorable relationships to the fertility complex can be expected, due to a shared synthesis pathway [18,19], it is important to clarify these relationships as well as to reveal possible common genetic backgrounds in order to avoid the loss of breeding progress in fertility traits, which was accomplished during the past decades. Therefore, this study aims to reveal antagonistic relationships as well as to identify genes or QTL with possible pleiotropic effects on boar taint and the analyzed endocrine parameters.

### 4.1. Descriptive Summary for Boar Taint and Endocrine Parameters

The descriptive data showed that the concentrations of AND and SKA in fat were, on average, greater in LR (1653.92 ng/g for AND, 219.48 ng/g for SKA) than in LW (1223.45 ng/g for AND and 93.26 ng/g for SKA). A reason for that could be the leaner carcass composition of LW pigs in comparison to LR pigs [27]. By application of the strictest thresholds of 150 ng/g for SKA and 1500 ng/g for AND, 23.1% of all LR and 4.7% of all LW boars would be classified as conspicuous.

For the hormone concentrations, the number of analyzed LW animals was lower (n = 286) than LR animals (n = 710) (Table 2), which resulted from the limited availability of purebred LW animals. Consequently, the results, especially for LW animals, estimated parameters have to be interpreted with caution, as can also be seen from the high standard errors of the estimators.

The mean CORT concentrations in this study are in a range between 28.31 ng/mL and 38.47 ng/mL. Comparing CORT concentrations to references is not recommendable as CORT is a hormone where the presence is highly influenced by many factors, e.g., the individual animal, breed, or the circadian rhythm, which cannot be expressed by a single measurement [28]

The mean TEST concentration was 11.82 ± 9.53 ng/mL for LR boars and 8.17 ± 7.53 ng/mL for LW boars. This is higher than the reported values of Colenbrander et al. [29], who measured approximately 4 ng/mL for a Dutch LR × Yorkshire cross at a similar age. Nevertheless, the increasing TEST concentration after this age has been described by Colenbrander et al. [29] as a consequence of increasing activity in the testis. Newer studies have shown TEST concentrations in a range of of 0.73 to 3.06 ng/mL in Duroc boars depending on the season [30] or concentrations of 6.6 ng/mL in a Pietrain × LR cross [31]. The average TEST concentration of females was 0.49 ± 1.66 in LR and 0.15 ± 0.12 in LW. The high number of females with a TEST concentration under the sensitivity threshold of 0.08 ng/mL underlines the hypothesis that the female animals in this study were not in estrus yet.

The average EST concentrations of 1.58 ± 1.50 ng/mL in LR and 0.97 ± 1.15 in LW were greater in the male animals than in the female animals (0.26 ± 0.26 ng/mL in LR, 0.21 ± 0.15 ng/mL in LW). The high concentration in male animals can be explained due to the fact, that boars produce high amounts of estrogens in the testis compared to other animals [32]. Additionally, the blood samples of females were collected in a prepubertal age. Under consideration of their PROG profile, it can be assumed that these gilts were not yet in estrus. Therefore, female animals have lower EST concentrations than the boars (Table 2).

The mean LH concentrations in this study were higher in females (7.03 ng/mL ± 4.27 in LR, 7.20 ng/mL ± 2.44 ng/mL in LW) than in males (2.81 ng/mL ± 1.54 in LR, 2.59 ng/mL ±1.03 in LW). For FSH, the concentrations of male and female animals did only slightly differ. Whereas female animals showed an average FSH concentration of 1335.54 ng/mL in LR and 1355.22 ng/mL in LW, male animals showed FSH concentrations of 1402.29 ng/mL in LR and 1440.00 ng/mL in LW.

PROG was excluded from further analysis, as there were too many values in the assay under the sensitivity threshold of 0.14 ng/mL. An explanation for the high number of animals under this threshold could be their age. Because PROG was earlier described as an endocrine indicator for the number of ovulations in prepubertal gilts [33], animals in our study may not have reached this hormonal status of puberty, as they were too young, with an average age of 126 days for females and 164 days for males. Additionally, studies dealing with the physiological background of PROG are often performed as a challenge study, during the estrous cycle or in pregnant or sows or gilts [34,35,36]. These studies cannot be used as references, as PROG during pregnancy plays another role than in prepubertal pigs.

### 4.2. Variance Component Estimation

The results for the h^2^ for boar taint compounds AND (h^2^ = 0.52 in LR, h^2^ = 0.44 in LW) and SKA (h^2^ = 0.40 in LR, h^2^ = 0.34 in LW) are in accordance with previous reported ranges of 0.25 to 0.88 for AND and 0.19 to 0.54 for SKA [10,37,38,39]. The genetic correlation between AND and SKA was r_g_ = 0.42 in LR and r_g_ = 0.57 in LW. In LR, this result is close to the previously reported r_g_ of 0.35 and 0.36 [10,11] and it is comparable to the results from our previous study, which represents a subset of this dataset (r_g_ = 0.29 in LR, r_g_ = 0.41 in LW) [13]. The physiological point of view for this relationship is described by Doran et al. [14] who described that SKA metabolism is inhibited by high AND concentrations in hepatocytes. Therefore, an increasing AND concentration leads to an increasing SKA concentration, which is reflected by the genetic correlations that are estimated in this study.

A few studies have already been conducted on the genetic relationship between boar taint and TEST and/or EST, as the common synthesis pathway for boar taint compounds and sex steroid hormones has been well known for a long time [15,40,41]. However, as endocrine parameters are underlying a regulatory network, it is important to not only focus on TEST or EST, but also reveal the relationships of boar taint to regulatory hormones like LH, or FSH.

Regarding the results presented in Table 3, it should be taken into account, that individual variation of these hormone concentrations is very high and the standard error of both h^2^ and r_g_ limits the significance of our study.

Moderate h^2^ of 0.35 for CORT in LW is in accordance with what has been reported in the literature of Larzul et al. [42], who have reported a h^2^ of 0.36 for a baseline of CORT in purebred LW. A study on Swiss LR showed h^2^ between 0.40 and 0.70, depending on the used model [43], which is higher than the h^2^ of 0.11 that was estimated for LR animals in this study. The genetic relationship between boar taint compounds and CORT was moderately negative in LR with a r_g_ = −0.18 for AND and SKA and −0.21 for SKA and CORT. Therefore, breeding against AND or SKA could result in a higher CORT concentration in LR animals.

The h^2^ for TEST differed between the breeds. The moderate h^2^ of 0.23 in LW is more comparable to the reported values for purebred Duroc (DU) (h^2^ = 0.19) and a DU x LW cross (h^2^ = 0.29) [44]. This study has also reported a high r_g_ between AND and TEST of 0.75 in the purebred boars [44], which we can generally confirm in LR (r_g_ = 0.62), whereas the r_g_ in LW was higher in this study (r_g_ = 0.93). These findings are in accordance with our expectations, because the synthesis pathway of AND is along with the sex steroid synthesis, where testosterone shows up as a precursor of AND [9,18]. Similar values were expected for r_g_ between SKA and TEST due to the moderate to high r_g_ between AND and SKA. In LR, r_g_ between AND and SKA was very high (r_g_ = 0.83) and comparable with the results of the study conducted by Parois et al. [44] (r_g_ = 0.71), whereas the r_g_ in LW was lower (r_g_ = 0.27). Because TEST has been reported to be essential for spermatogenesis and male fertility in general [45,46], breeding against AND and/or SKA would have clear unfavorable consequences on the concentration of TEST in purebred LR and LW animals.

EST is not only one of the most important estrogens for female fertility, as reflected by Grindflek et al. [41]; it is also very important for sexual behavior in boars. Genetic correlations between AND and EST have been reported in several studies in a range between 0.42 to 0.93, as summarized by Moe et al. [15], where r_g_ in this study was 0.49 for LR and 0.46 for LW. In contrast to that, r_g_ between SKA and EST in LR was very high in this study (r_g_ = 0.95) and low in LW (r_g_ = 0.03) when compared to the literature references of 0.29 to 0.53, as reviewed by Moe et al. [15]. Nevertheless, it should be noted that the sample size for LW animals in this study was comparably low to the LR sample size, which has an impact on the estimates and the standard errors.

The genetic correlation between FSH and AND was 0.30 in LR and LW, respectively, which indicates that breeding against AND would result in lower FSH concentrations. This could have relevant undesirable consequences, as has been shown by Wise et al. [47] in Meishan pigs that were used to improve the fertility of European pig populations. Within this breed, the authors have shown that there is a negative relationship between FSH concentration in boars and increased litter size in sows. Regarding the genetic relationship between FSH and SKA, the risk of reduced fertility by breeding against SKA is small, because the r_g_ is close to zero in LW (r_g_ = 0.01) and even favorably expressed in LR (r_g_ = −0.14).

### 4.3. GWAS

In this study, univariate GWAS for the analyzed hormones TEST, CORT, EST, LH, and FSH was performed for three different clusters: Ho_LR_C (commercial LR population), Ho_LW_ (LW population from herd book and commercial breeding organization), and Ho_LR_H (herd book LR population). This association analysis revealed nine markers in cluster Ho_LR_C, 19 (4) markers in cluster Ho_LW_ and four markers in cluster Ho_LR_H, which were found to be chromosome-wide (genome-wide) significantly associated with one of the analyzed hormones.

#### 4.3.1. GWAS Hormones

For CORT in Ho_LR_C, two intron variants were identified around 144.8 Mb on SSC 2 belonging to the gene Nuclear Receptor Subfamily 3 Group C Member 1 (*NR3C1*). This gene is known as a candidate gene for affecting the regulation of the hypothalamic-pituitary-adrenal axis (HPA axis) in pigs [48,49]. Additionally, a polymorphism of this gene was later identified with variations in plasma cortisol levels in purebred LR and LW populations as well as in a Pietrain × (LW × LR) crossbred [49].

For Ho_LW_, a region around 115.6 Mb on SSC 7 contained significantly associated variants with CORT. One of them is an intron variant of the gene DEAD-Box Helicase 24 (*DDX24*). Although this gene is not further investigated in pig, the members of this gene family are potentially involved in, e.g., spermatogenesis in humans [50]. Based on this information, *DDX24* can be regarded as a possible candidate gene for TEST concentration in pigs. The previously reported associations with CORT and variations in CORT concentrations in a LW × Meishan cross by Désautés et al. [51] are located on SSC 7 at 149 Mb and 156 Mb. Additionally, Ponsuksili et al. [52] identified two regions on SSC 7 at 123.2 Mb and 85.9 Mb to be significantly associated with plasma CORT concentration in a Pietrain × (LW × LR) crossbred. A marker on SSC 18 at 33.3 Mb was identified in this study for Ho_LR_H, but the position is not in accordance with results that were described by Désautés et al. [51].

Within Ho_LR_C and Ho_LW_, GWAS for TEST revealed six and eight chromosome-wide associations, respectively. For Ho_LR_C, two regions were identified on SSC 1 around 15 Mb and 150.6 Mb. The first region contains two intergenic variants and two intron variants of the gene A-Kinase Anchoring Protein 12 (*AKAP12*). Until now, no specific function of this gene is known in the porcine organism. Two additional markers were identified on SSC 7 at 106.5 Mb and 113.1 Mb in Ho_LR_C affecting TEST. Because the first marker is an intergenic variant and the second one is an unknown variant, no statement can be made due to their relevance for TEST. The region on SSC 7 at 71 Mb described by Ren et al. [53] in a F2 LW × Erhualian intercross was not confirmed. For the Ho_LW_ cluster, a region between 33.9 Mb to 37.0 Mb on SSC 16 was identified to be significantly associated with TEST. This region contains an upstream gene variant of Granzyme K (*GZMK*). Additionally, this region comprises four introns, two intergenic variants, and one unknown variant. None of these variants are in genes that seem to play a role in TEST synthesis or regulation. Nevertheless, one intron variant is located in the interleukin−6 receptor subunit beta (*IL6ST*). Variations in this gene or members of his family could be possibly involved in alterations in the expression of these receptors in porcine follicular fluid during atresia [54]. Grindflek et al. [55] identified QTL for TEST in Norwegian LR and Duroc on the same chromosome, at 21 to 24 Mb. Previously identified regions on SSC 3 [55], SSC 7, or SSC 13 [53] for TEST could not be confirmed in this study.

GWAS for EST only showed chromosome-wide significant associations with two markers in cluster Ho_LR_H. One marker was located on SSC 6 at 69.5 Mb and it was identified as an intron variant of the Solute Carrier Family 2 Member 5 (*SLC2A5*). This gene is involved in transporting fructose during pregnancy from uterus to conceptus [56]. Additionally, it has been hypothesized by Steinhauser et al. [56] that an increasing concentration of PROG results in a higher expression of *SLC2A5*. In conclusion, it cannot be hypothesized that *SLC2A5* is a specific candidate gene for EST, but for fertility in general. Associations for EST on SSC 1, 13, or 15, as reported by Grindflek et al. [55], could not be confirmed in our study.

For LH, one marker was found to be chromosome-wide significantly associated in Ho_LR_H. This marker an intron variant of Phosphatase And Actin Regulator 1 (*PHACTR1*) was located on SSC 7 at 9.5 Mb. The specific function of this gene in pigs is still unknown. In humans, a homologue of this gene is associated with the risk for coronary artery diseases [57]. In a broad sense, this gene function can be linked to robustness, but not to maternal or paternal fertility characteristics.

FSH was associated with at least one marker when analyzing the clusters Ho_LR_C and Ho_LW_, respectively. For Ho_LR_C, the marker was an intergenic variant on SSC 10. For Ho_LW_, an intron variant on SSC 7 at 117.9 Mb was identified, lying in the gene VRK Serine/Threonine Kinase 1 (*VRK1*). This gene is involved in the phosphate metabolism at least in Berkshire and Korean native breeds [58], but, until now, it is not possible to relate this gene function to any kind of fertility influencing metabolism.

In general, GWAS of hormone data showed significant regions that differed per trait and cluster. No overlapping regions were found in the analysis of endocrine parameters, although a region on SSC 7 contained significant associations for CORT in Ho_LW_, TEST in Ho_LR_C, and FSH in Ho_LW_ in adjacent regions between 113.1 Mb to 117.9 Mb.

#### 4.3.2. GWAS Boar Taint Cluster BT_LR_H

A previous study already performed association studies for a LR and LW population from a commercial breeding organization [13]. This study showed genome-wide associations with AND in LR on SSC 5 and in LW on SSC 17. For SKA, GWAS showed significant associations in both breeds for the region around 141 Mb on SSC 14. Furthermore, a region on SSC 6 at 0.3 Mb to 0.4 Mb was significantly associated with SKA in LR [13].

The results of this analysis could be partially confirmed by the GWAS in this study, which was performed in the LR herd book dataset BT_LR_H. GWAS was not performed within the LW herd book dataset (BT_LW_H) because of the small sample size.

GWAS for cluster BT_LR_H revealed 13 markers, which were found to be chromosome-wide significantly associated with SKA. For AND, no significant associated markers were found. For SKA, 12 of the 13 identified markers are located in a region on SSC 14, which ranged from 140.5 Mb to 141.3 Mb. Although none of these markers are located in genes, which are further investigated in pigs, the identified region was identified as the promotor region of the Cytochrome P450 Family 2 Subfamily E Member 1 (*CYP2E1*) gene, which is well known to be involved in SKA metabolism from several previous studies [15,59,60,61,62]. Nine of these 12 markers were also found to be significantly associated with SKA in the BT_LW_C cluster in the previous study [13], which enhances the importance of this region for a genetic background for SKA.

Knowledge about overlapping gene regions is of particular interest regarding the key question of whether selection against boar taint has negative consequences on sexual hormones. However, none of the identified regions for endocrine parameters in this study are located in regions that were identified for AND or SKA or reproduction traits in our previous study [13]. Consequently, clear genomic indicators that provide evidence of an antagonistic relationship between boar taint and fertility were not found in this study.

## 5. Conclusions

In conclusion, the results showed contrary directions regarding the possible unfavorable relationships between boar taint compounds and reproduction hormones in both of the breeds. However, in the hormones EST and TEST, which are well-known for their importance for female and male fertility, r_g_ are showing consistent unfavorable relationships among both breeds regarding breeding against AND. These results confirm the physiologically expected relationship also on the genetic level.

GWAS could not identify regions with pleiotropic effects on boar taint and EFP, but it enhances the importance of the identified region on SSC 14 for SKA. The performed GWAS for endocrine parameters revealed possible candidate genes for fertility. A region on SSC 7 between 113.1 Mb to 117.9 Mb showed pleiotropic potential for CORT, TEST, and FSH, which should be further analyzed while using multivariate approaches. Although a high h^2^ of AND and SKA seems to be promising regarding breeding against boar taint, selection should generally be handled with care, as a deterioration of breeding progress in reproduction traits should be avoided.

## Figures and Tables

**Table 1:** Number of animals and markers per trait and cluster.

Trait	Cluster	Number of Animals	Number of Markers
AND	BT_LR_C *	1293	38,411
	BT_LW_C *	1317	39,302
	BT_LR_H	735	43,644
SKA	BT_LR_C *	1293	38,411
	BT_LW_C *	1317	39,302
	BT_LR_H	735	43,644
CORT	Ho_LR_C	254	40,176
	Ho_LW_	271	40,972
	Ho_LR_H	434	44,095
TEST	Ho_LR_C	252	40,176
	Ho_LW_	267	40,972
	Ho_LR_H	423	44,095
EST	Ho_LR_C	251	40,176
	Ho_LW_	265	40,972
	Ho_LR_H	415	44,095
LH	Ho_LR_C	254	40,176
	Ho_LW_	272	40,972
	Ho_LR_H	417	44,095
FSH	Ho_LR_C	254	40,176
	Ho_LW_	272	40,972
	Ho_LR_H	417	44,095

* = results are shown in previous study [13], HO_LR_C = hormone cluster Landrace from a commercial breeding organization, HO_LW = hormone cluster Large White from a commercial breeding organization, HO_LR_H = hormone cluster Landrace from a herd book organization, AND = log-transformed androstenone, SKA = log-transformed skatole, CORT = log-transformed cortisol, TEST = log-transformed testosterone, EST = log-transformed estradiol, LH = log-transformed luteinizing hormone, and FSH = log-transformed follicle-stimulating hormone. 2.6. GWAS.

**Table 2:** Descriptive statistics of the analyzed traits (log-transformed).

Trait	Sex	Landrace		Large White	
		N	Mean ± SD	N	Mean ± SD
Age	female	353	4.85 ± 3.44	138	4.83 ± 2.05
	male	357	5.11 ± 2.49	148	5.09 ± 1.58
CORT	female	353	3.34 ± 2.59	137	3.49 ± 2.71
	male	357	3.53 ± 2.84	148	3.65 ± 3.03
	male + female	710	3.44 ± 2.75	285	3.58 ± 2.91
TEST	female	344	−0.71 ± 0.51	133	−1.90 ± −2.12
	male	353	2.44 ± 2.25	148	2.08 ± 2.02
	male + female	697	1.81 ± 2.17	281	1.46 ± 1.91
EST	female	340	−1.35 ± −1.35	132	−1.56 ± −1.90
	male	346	0.46 ± 0.41	147	−0.03 ± 0.14
	male + female	686	−0.07 ± 0.24	279	−0.50 ± −0.08
LH	female	343	1.95 ± 1.45	138	1.97 ± 0.89
	male	357	1.03 ± 0.43	148	0.95 ± 0.03
	male + female	700	1.59 ± 1.34	286	1.58 ± 1.10
FSH	female	348	7.20 ± 6.64	138	7.21 ± 6.13
	male	357	7.25 ± 6.92	148	7.27 ± 6.64
	male + female	705	7.22 ± 6.80	286	7.24 ± 6.46
AND	male	2136	7.41 ± 7.32	1639	7.11 ± 7.01
SKA	male	2136	5.40 ± 5.53	1639	4.54 ± 4.88

Age = age at sampling in days, CORT = log-transformed cortisol, TEST = log-transformed testosterone, EST = log-transformed estradiol, LH = log-transformed luteinizing hormone, FSH = log-transformed follicle-stimulating hormone, AND = log-transformed androstenone, SKA = log-transformed skatole.

**Table 3:** h^2^, r_g_ and r_p_ of boar taint compounds and hormone concentrations in Landrace (LR) and Large White (LW).

Trait	Breed	AND		SKA		CORT		TEST		EST		LH		FSH	
AND	LR	0.52	(0.07)	0.42	(0.11)	−0.18	(0.28)	0.62	(0.91)	0.49	(0.33)	0.11	(0.20)	0.30	(0.19)
	LW	0.44	(0.07)	0.57	(0.12)	0.08	(0.26)	0.83	(0.34)	0.46	(0.27)	0.32	(n.E.)	0.30	(0.25)
SKA	LR	0.34		0.40	(0.06)	−0.21	(0.30)	0.93	(n.E.)	0.95	(0.40)	−0.16	(0.21)	−0.14	(0.20)
	LW	0.27		0.34	(0.07)	0.38	(0.30)	0.27	(0.36)	0.03	(0.30)	0.45	(n.E.)	0.01	(0.29)
CORT	LR	−0.01		−0.01		0.11	(0.08)	−0.35	(n.E.)	0.01	(0.60)	−0.27	(0.42)	0.03	(0.37)
	LW	0.02		0.02		0.35	(0.17)	−0.03	(0.48)	−0.26	(0.41)	−0.42	(n.E.)	−0.58	(0.38)
TEST	LR	0.32		0.28		−0.02		0.03	(0.08)	0.89	(n.E.)	0.11	(0.86)	0.11	(0.72)
	LW	0.52		0.29		0.12		0.23	(0.18)	0.58	(0.34)	0.01	(n.E.)	−0.06	(0.45)
EST	LR	0.36		0.41		0.06		0.65		0.09	(0.08)	−0.22	(0.42)	−0.13	(0.42)
	LW	0.47		0.27		0.04		0.76		0.42	(0.25)	−0.04	(n.E.)	0.17	(0.39)
LH	LR	0.10		−0.03		0.07		−0.32		−0.16		0.28	(0.10)	0.91	(0.17)
	LW	0.15		0.12		0.07		0.24		0.15		0.04	(0.19)	0.66	(n.E.)
FSH	LR	0.14		0.07		−0.08		0.14		0.17		0.42		0.28	(0.09)
	LW	0.20		0.03		−0.04		0.15		0.13		0.52		0.37	(0.17)

h^2^ (± standard error) on the diagonal, r_p_ = phenotypic correlation under the diagonal, r_g_ = genetic correlation above the diagonal, AND = log-transformed androstenone, SKA = log-transformed skatole, CORT = log-transformed cortisol, TEST = log-transformed testosterone, EST = log-transformed estradiol, LH = log-transformed luteinizing hormone, FSH = log-transformed follicle-stimulating hormone.

**Table 4:** Genetic correlation (±SE) between male and female hormone concentrations.

	CORT	TEST	EST	FSH	LH
Genetic correlation	0.80 (n.E.)	0.91 (n.E.)	0.42 (n.E.)	0.75 (±0.59)	0.88 (±0.43)

CORT = log-transformed cortisol, TEST = log-transformed testosterone, EST = log-transformed estradiol, FSH = log-transformed follicle-stimulating hormone, LH = log-transformed luteinizing hormone.

**Table 5:** Chromosome wide significant marker in clusters after Bonferroni correction (*p* < 0.05).

Trait	Cluster	SNP Name	SSC	Position (Mb)	MAF	Variant	Variance by SNP	SNP Effect (± SE)	Effect Allele	Gene Symbol
CORT	Ho_LR_C	ALGA0106239	2	144.8	0.15	Intron	0.088	−0.314 (±0.06)	A	NR3C1
		DRGA0017574	2	144.8	0.15	Intron	0.088	−0.314 (±0.06)	T	NR3C1
		FBF0920 *	7	115.2	0.24	-	0.087	0.274 (±0.05)	-	-
		CASI0004483 *	7	115.3	0.17	Intron	0.115	0.374 (±0.06)	A	DDX24
		ALGA0045097	7	115.6	0.51	Intron	0.071	0.202 (±0.04)	A	alpha-1-antiproteinase-like
		FBF0965	7	115.6	0.51	-	0.069	0.199 (±0.04)	-	-
	Ho_LW_	FBF0971 *	7	115.6	0.18	-	0.123	0.375 (±0.06)	-	-
		H3GA0023283	7	115.6	0.72	Intron	0.077	−0.233 (±0.05)	G	SERPINA11
		FBF0974	7	115.6	0.72	-	0.077	−0.233 (±0.05)	-	-
		MARC0043760*	7	115.6	0.78	Intron	0.109	−0.326 (±0.06)	G	SERPINA11
		FBF0973	7	-	0.71	-	0.076	−0.231 (±0.05)	-	-
	Ho_LR_H	DIAS0003615	18	33.3	0.35	n.m.	0.038	−0.137 (±0.03)	G	-
TEST	Ho_LR_C	ASGA0001286	1	14.9	0.57	Intron	0.081	0.183 (±0.04)	A	AKAP12
		DRGA0000172	1	15.0	0.57	Intron	0.081	0.183 (±0.04)	T	AKAP12
		ALGA0001286	1	15.0	0.43	intergenic	0.082	−0.184 (±0.04)	C	-
		ASGA0001297	1	150.6	0.43	intergenic	0.082	−0.184 (±0.04)	C	-
		ALGA0044414	7	106.5	0.04	intergenic	0.084	0.487 (±0.10)	T	-
		INRA0028035	7	113.1	0.04	n.m.	0.084	0.487 (±0.10)	C	-
	Ho_LW_	ASGA0073034	16	33.9	0.40	intergenic	0.074	0.250 (±0.05)	G	-
		ASGA0073036	16	34.0	0.40	intergenic	0.074	0.250 (±0.05)	A	-
		MARC0056521	16	34.2	0.39	UGV	0.079	0.263 (±0.06)	G	Granzyme K
		ALGA0116942	16	34.7	0.37	Intron	0.069	0.242 (±0.05)	C	PLPP1
		ASGA0073065	16	35.0	0.37	Intron	0.068	0.241 (±0.05)	G	IL31RA
		ALGA0090291	16	35.1	0.37	Intron	0.068	0.238 (±0.05)	T	IL6ST
		ASGA0096589	16	35.3	0.37	Intron	0.068	0.241 (±0.05)	C	ANKRD55
		SIRI0000852	16	37.0	0.41	n.m.	0.078	0.249 (±0.05)	A	-
EST	Ho_LR_H	MARC0051573	6	69.5	0.39	Intron	0.044	−0.274 (±0.06)	T	SLC2A5
		ASGA0075694	17	19.6	0.10	NCTEV	0.048	0.445 (±0.10)	T	ENSSSCG00000048560
LH	Ho_LR_H	ALGA0038510	7	9.5	0.21	Intron	0.043	−0.159 (±0.04)	A	PHACTR1
FSH	Ho_LR_C	MARC0079871	10	58.1	0.01	intergenic	0.069	0.628 (±0.14)	C	-
	Ho_LW_	DBNP0002208	7	117.9	0.19	Intron	0.065	0.146 (±0.03)	C	VRK1
SKA	BT_LR_H	M1GA0020074	14	140.5	0.69	UGV	0.035	−0.233 (±0.04)	A	LRRC27
		MARC0028756	14	140.6	0.69	Intron	0.035	−0.233 (±0.04)	A	LRRC27
		M1GA0020080	14	140.6	0.27	UGV	0.035	0.244 (±0.05)	C	LRRC27, PWWP2B
		M1GA0020121	14	140.9	0.27	3’PUTR	0.035	0.242 (±0.05)	T	CFAP46
		M1GA0020138	14	141.0	0.27	DGV	0.033	0.238 (±0.05)	G	ENSSSCG00000047411
		ALGA0083389	14	141.1	0.27	DGV	0.034	0.239 (±0.05)	T	ADGRA1
		INRA0048622	14	141.1	0.27	Intron	0.034	0.239 (±0.05)	T	KNDC1
		ASGA0068302	14	141.2	0.27	UGV	0.034	0.239 (±0.05)	G	ADAM8, TUBGCP2
		H3GA0043620	14	141.2	0.27	intergenic	0.034	0.239 (±0.05)	G	-
		ASGA0068308	14	141.3	0.27	Intron	0.034	0.239 (±0.05)	G	CALY
		H3GA0043634	14	141.3	0.27	3’PUTR	0.034	0.239 (±0.05)	T	ECHS1
		H3GA0043632	14	141.3	0.27	Intron	0.034	0.239 (±0.05)	A	MTG1
		INRA0048614	14	152.9	0.27	n.m.	0.034	0.239 (±0.05)	G	-

Ho_LR_C = hormone dataset Landrace from a commercial breeding organization, Ho_LW_ = hormone dataset Large White from commercial and herd book organizations, Ho_LR_H = hormone dataset Landrace from herd book organizations, BT_LR_H = boar taint dataset Landrace from herd book organizations, CORT = log-transformed cortisol, TEST = log-transformed testosterone, EST = log-transformed estradiol, LH = log-transformed luteinizing hormone, FSH = log-transformed follicle-stimulating hormone, SKA = log-transformed skatole, SSC = Sus Scrofa Chromosome, MAF = Minor allele frequency, * = also genome-wide significant, n.m. = not mapped, UGV = upstream gene variant, NCTEV = non coding transcript exon variant, 3’PUTR = 3’ prime untranslated region, DGV = downstream gene variant. Genome-wide association analysis (GWAS) for TEST revealed six chromosome-wide significant markers for Cluster Ho_LR_C and eight chromosome-wide significant markers for Cluster Ho_LW_. Two markers for cluster Ho_LR_C are located on SSC 7 at 106.5 Mb and 113.1 Mb and were an intergenic and a not mapped variant. Furthermore, four markers were located around 150 Mb on SSC 1 containing two introns and two intergenic variants. For Cluster Ho_LW_, seven markers were located in a region from 33.9 Mb to 35.3 Mb on SSC 16 and contained four intron variants, two intergenic variants, and one upstream gene variant (UGV). The last variant was located at 37.0 Mb and it was not mapped.

## Data Availability

Restrictions apply to the availability of these data. Data was obtained from members of the Association for Bioeconomy Research (FBF e.V.) and are available from Sebastian Klein or Ernst Tholen with the permission of the particular member.

## Supplementary Materials

The following tables are available online at https://www.mdpi.com/2076-2615/11/1/231/s1, Table S1: Descriptive statistics of the analyzed traits (raw values). Table S2: Chromosome wide significant marker in clusters after Bonferroni correction (*p* < 0.05). Table S3: Adjacent regions and possible interesting genes in a window of +/−1 Mb.

## Abbreviations

3’PUTR	3’prime untranslated region
AKAP12	A-Kinase Anchoring Protein12
AND	Androstenone
BT_LR_C	Boar taint cluster Landrace, commercial breeding organization
BT_LR_H	Boar taint cluster Landrace, herd book organizations
BTLWC	Boar taint cluster Large White, commercialbreeding organization
BTLWH	Boar taint cluster Large White, herd book organizations
C	Commercial breeding organization
CORT	Cortisol
CYP2E1	Cytochrome P450 Family 2 Subfamily E Member 1
DDX24	DEAD-Box Helicase 24
DGV	Downstream gene variant
EFP	Endocrine fertility parameters
EST	17-β estradiol
FSH	Follicle-stimulating hormone
GC	Genomic Control
GWAS	Genome-wide association analysis
GZMK	Granzyme K
H	Herd book organizations
h^2^	Heritability
Ho_LR_C	Hormone cluster Landrace, commercial breeding organization
Ho_LR_H	Hormone cluster Landrace, herd book organizations
Ho_LW_	Hormone cluster Large White from all organizations
HPA axis	Hypothalamic-pituitary-adrenal axis
IL6ST	interleukin-6 receptor subunit beta
intra-CV	Intra-variation coefficient
kg	Kilogram
LH	Luteinizing hormone
LR	Landrace
LW	Large White
MAF	Minor allele frequency
ml	Milliliter
n.m.	Not mapped
NCTEV	non coding transcript exon variant
ng	Nanogram
NR3C1	Nuclear Receptor Subfamily 3 Group C Member 1
PHACTR1	Phosphatase And Actin Regulator 1
PROG	Progesterone
QTL	Quantitative trait locus/loci
r_g_	Genetic correlation
r_p_	Phenotypic correlation
SIDA-HSPM-GC/MS	Stable isotope dilution analysis-headspacesolid-phase microextraction-gas chromatography/mass spectrometry
SKA	Skatole
SLC2A5	Solute Carrier Family 2 Member 5
SNPs	Single nucleotide polymorphism(s)
SSC	*Susscrofachromosome*
TEST	Testosterone
UGV	Upstream gene variant
VCE	Variance component estimation
VRK1	VRK Serine/Threonine Kinase 1
